# Comparative Analysis of Physicochemical Properties and Storability of a New Citrus Variety, Yellowball, and Its Parent

**DOI:** 10.3390/plants12152863

**Published:** 2023-08-03

**Authors:** Dong-Shin Kim, Sung-man Jeong, Seong-Ho Jo, Saoraya Chanmuang, Sang Suk Kim, Suk Man Park, Su Hyun Yun, Seung-Gab Han, Jeong-Yong Cho, Inhae Kang, Hyun-Jin Kim

**Affiliations:** 1 Institute of Animal Medicine, Gyeongsang National University, Jinju 52828, Republic of Korea; feel567@naver.com; 2 Division of Applied Life Science (BK21 Four), Gyeongsang National University, Jinju 52828, Republic of Korea; wjdtjdaks97@naver.com (S.-m.J.); dkf32@naver.com (S.-H.J.); 3 Institute of Agriculture and Life Science, Gyeongsang National University, Jinju 52828, Republic of Korea; c.saoraya@gmail.com; 4 Citrus Research Institute, National Institute of Horticultural & Herbal Science, Rural Development Administration, Seogwipo 63607, Republic of Korea; sskim0626@korea.kr (S.S.K.); babau2000@korea.kr (S.M.P.); yunsh@korea.kr (S.H.Y.); skhan@korea.kr (S.-G.H.); 5 Department of Food Science and Technology, Chonnam National University, Gwangju 61186, Republic of Korea; jyongcho17@connam.ac.kr; 6 Department of Food Science and Nutrition, Jeju National University, Jeju 63243, Republic of Korea; inhaek@jejunu.ac.kr; 7 Department of Food Science and Technology, Gyeongsang National University, Jinju 52828, Republic of Korea

**Keywords:** citrus, metabolomics, sensory evaluation, storability, Yellowball

## Abstract

Although numerous citrus varieties have recently been developed to enhance their quality, information on their quality characteristics is limited. We assessed the quality characteristics of Yellowball, a novel citrus variety, by evaluating its appearance, storability, sensory properties, functionality, and metabolite profiles and then comparing these characteristics with those of its parent varieties, Haruka and Kiyomi. The metabolite profiles between the citrus varieties differed significantly, resulting in distinct physicochemical and functional qualities. The storability of Yellowball was significantly increased compared with that of its parent varieties owing to its strong antifungal activity and unique peel morphology, including the stoma and albedo layers. While we did not investigate the volatile compounds, overall functional activities, and detailed characteristics of each metabolite, our data provide valuable insights into the relationship between citrus metabolites, peel morphology, physicochemical properties, and storability, and demonstrate the potential of Yellowball as a promising variety in the citrus industry.

## 1. Introduction

Citrus is one of the most essential non-climacteric fruit crops that are grown in more than 140 countries. In 2019, approximately 143,755,000 tons of citrus was produced worldwide [1]. These fruits are either consumed fresh or processed into various products, such as juice, jam, and vinegar, owing to their unique favorable taste, which is a result of the good balance between sugars, acidic compounds, and secondary metabolites [2]. Additionally, vitamins (vitamin A, ascorbic acid, and folate), minerals (Ca, P, K, Mg, Zn, and Se) secondary metabolites (phenolic compounds, limonoids, and carotenoids) have various functional properties, including antioxidant, anti-microbial, anti-inflammatory, anti-cancer, anti-obesity, and anti-diabetic activities [3,4,5]. However, the taste and functional qualities of citrus fruits are clearly different according to the variety, environmental growing conditions, and postharvest conditions [6,7]. In particular, the chemical profiles are considerably different between citrus varieties, including oranges, lemons, grapefruits, limes, mandarins, pomelos, kumquats, and tangelos [7,8].

In the past, more than 140 different genera and 1300 different citrus species, including the general varieties, have been reported [9], and are still extensively hybridized to improve their productivity, appearance, sugar/acid balance, flavor, functional compounds, and tolerance to abiotic and biotic stresses [10,11,12]. However, despite many attempts, the development of citrus varieties with qualities unique from their parent varieties is still a challenge [13].

Yellowball (*C. hybrid cv.* Yellowball) is a new citrus variety hybridized between Haruka (*C. tamuranua*) and Kiyomi (*C. unshiu × sinensis*). Kiyomi is a well-known variety with a better peelability than sweet orange (*C. sinensis*) and a superior aroma to satsuma (*C. unshiu*) [13]. Additionally, Kiyomi contributed to the development of more than 16 citrus varieties with unique features. such as less rind puffing, less alternate bearing, grenadine to red rind, richness in β-cryptoxanthin, and unique fruit shape [13]. Conversely, the use of Haruka in citrus breeding is limited. Few studies have reported on Haruka, including its hybrid origin and a novel Citrivirus isolate that was discovered in this variety [14,15]. Yellowball is a unique hybrid with characteristics distinct from conventional citrus varieties. However, current information regarding its quality characteristics is limited.

Therefore, to investigate the quality characteristics of Yellowball, we analyzed the appearance, soluble solids content (SSC), titratable acidity (TA), sensory quality, storability, metabolite profiles, and functional activities (antioxidant and anti-inflammatory activities) of Yellowball and compared these characteristics with those of its parent varieties, Haruka and Kiyomi. Additionally, we analyzed the correlations between metabolite profiles and these quality characteristics.

## 2. Results

### 2.1. Appearance

**Figure 1:**
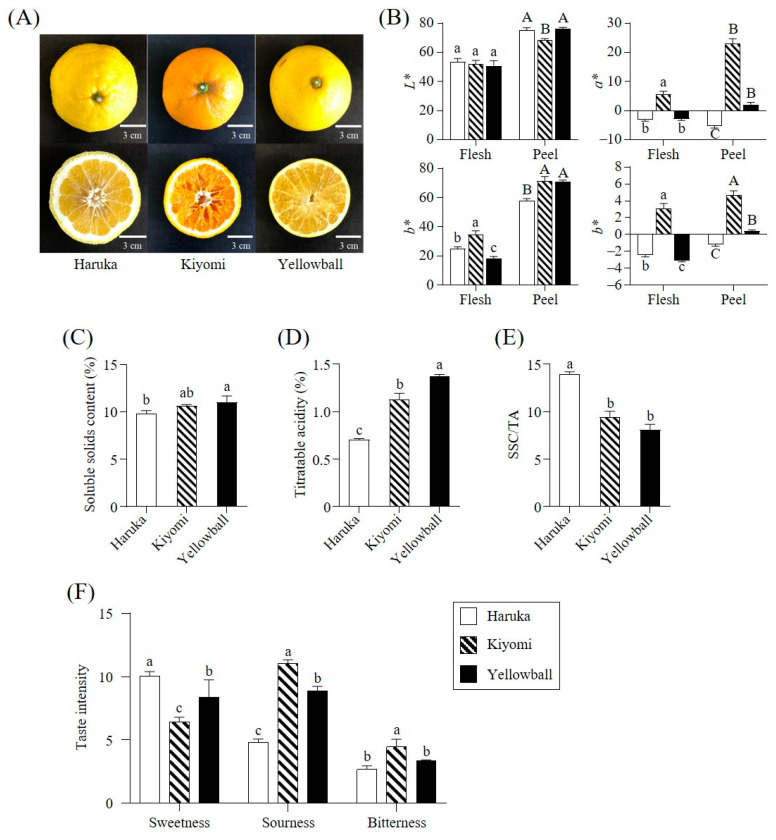
Appearance (**A**), color values (**B**), soluble solids content (SSC) (**C**), titratable acidity (TA) (**D**), SSC/TA ratio (**E**), and sensory analysis (**F**) of Haruka, Kiyomi, and Yellowball. Different letters in each bar indicate a significant difference as determined by Duncan’s test (*p* < 0.05). Intensity of each sensory quality was rated on a 15 cm line scale, labeled “very weak” and “very strong”, with 0.5 cm anchors on the left and right sides. L*, lightness; a*, redness; b*, yellowness.

The appearance characteristics of Yellowball were compared with those of Haruka and Kiyomi (Figure 1 and Table S1). The weight and size of Yellowball were similar to those of Kiyomi, but smaller than those of Haruka. The peel thicknesses were as follows: Yellowball (0.44 ± 0.03 cm) < Kiyomi (0.68 ± 0.12 cm) < Haruka (0.90 ± 0.09 cm).

The flesh and peel colors of Yellowball and Haruka were yellow, while those of Kiyomi were orange (Figure 1A). These results were confirmed by the color values, including L*, a*, b*, and the citrus color index (CCI) (Figure 1B). The a* (5.56 ± 1.10 for flesh; 22.97 ± 1.75 for peels) and CCI values (3.11 ± 0.57 for flesh; 4.71 ± 0.48 for peels) of Kiyomi were significantly higher than those of Yellowball (a*= −2.86 ± 0.50 for flesh and 2.10 ± 0.78 for peels; CCI = 3.08 ± 0.17 for flesh and 0.39 ± 0.15 for peels) and Haruka (a*= −3.20 ± 0.40 for flesh and −5.16 ± 0.83 for peels; CCI = −2.41 ± 0.25 for flesh and −1.18 ± 0.19 for peels) (*p* < 0.05).

### 2.2. SSC, TA, and Sensory Quality

The SSC of Yellowball flesh (11.03 ± 0.67%) was slightly higher than that of Haruka (9.83 ± 0.32%) (Figure 1C), while the TA of Yellowball (1.37 ± 0.02%) was 94% and 21% higher than that of Haruka (0.70 ± 0.01%) and Kiyomi (1.13 ± 0.06%), respectively (*p* < 0.05) (Figure 1D). The SSC/TA ratio was calculated based on these data. The SSC/TA ratio of Yellowball (8.1 ± 0.6) was lower than that of Haruka (14.0 ± 0.2) (*p* < 0.05) (Figure 1E).

The sensory quality analysis of the three varieties (Figure 1F) revealed that sweetness and sourness were the major tastes of Haruka, Kiyomi, and Yellowball, whereas bitterness was minor tastes. The sweetness (8.36 ± 1.38) and sourness (8.88 ± 0.35) of Yellowball showed intermediate intensities between those of Haruka (sweetness: 10.06 ± 0.34 and sourness: 4.79 ± 0.27) and Kiyomi (sweetness: 6.43 ± 0.37 and sourness: 11.03 ± 0.29), whereas the bitterness of Yellowball was similar to that of Haruka (*p* < 0.05).

### 2.3. Storability, Antifungal Activity, Water Vaper Permeability (WVP), and Peel Morphology

**Figure 2:**
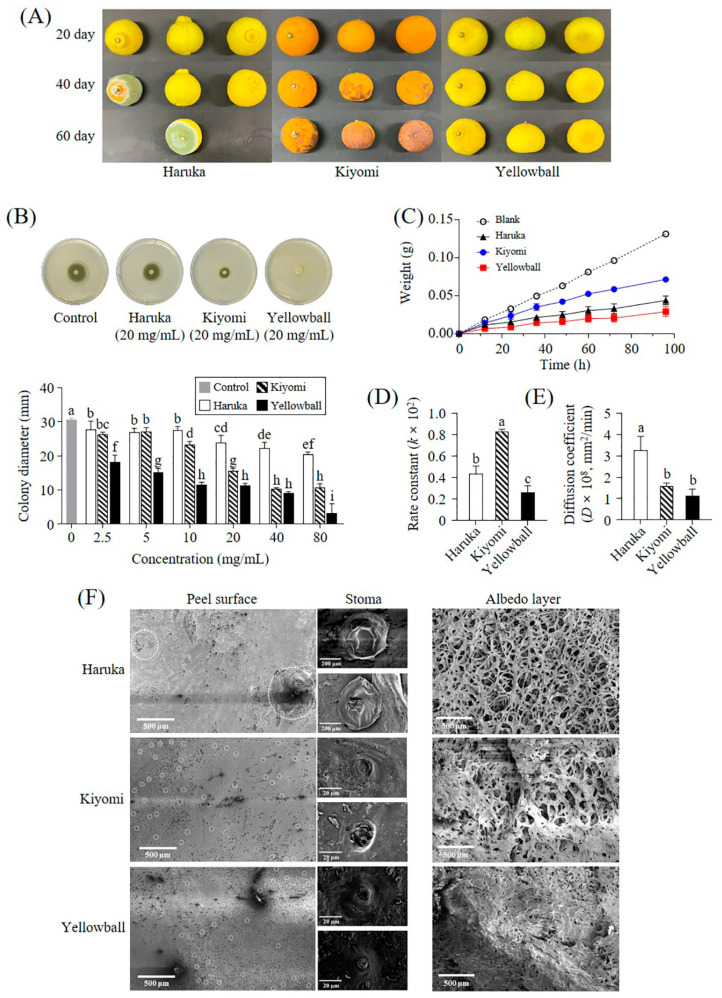
Storability and peel characteristics of Haruka, Kiyomi, and Yellowball. Appearance changes in Haruka, Kiyomi, and Yellowball during 60-day storage at room temperature (**A**). Anti-fungal activity of peel extract (**B**). Water vapor permeability (WVP) test results and weight of water vapor passing through the peel (**C**). Rate constant (**D**) and diffusion coefficient of WVP (**E**). Scanning electron microscopy (SEM) images of peels (**F**). Different letters in each bar indicate a significant difference as determined by Duncan’s test (*p* < 0.05). Rate constant and diffusion coefficient values were calculated using the first-order kinetic model and Fick’s second law of diffusion, respectively. White dotted circles on SEM images of the peel surface indicate the stoma.

The storability of the three citrus varieties was evaluated during the 60-day storage period (Figure 2A and Figure S1). After 35 d, Haruka began to decay, and the Kiyomi peels began to dry and turn brown. Except for partial drying, the appearance of Yellowball remained unchanged for 60 d. To determine the factors involved in storability, the antifungal activity (Figure 2B and Figure S2) and WVP (Figure 2C–E) of the peels of the three citrus varieties were measured. Yellowball showed stronger inhibitory activity against *Penicillium digitatum* than Haruka and Kiyomi (*p* < 0.05). *P. digitatum* did not grow in Yellowball and Kiyomi extract with concentrations above 10 mg/mL and 40 mg/mL, respectively, whereas no antifungal activity was observed at all tested Haruka extract concentrations (2.5–80 mg/mL).

The water permeability of the peels was significantly different between the three citrus varieties (Figure 2C). The amount of water vapor that passed through the Yellowball peels (0.029 ± 0.006 g) up to 96 h was significantly lower than that in Haruka (0.044 ± 0.006 g) and Kiyomi (0.072 ± 0.004 g), resulting in a lower rate constant (0.26 ± 0.06 × 10^2^) and diffusion coefficient (1.12 ± 0.33 mm^2^/min) in Yellowball than in Haruka (*k*: 0.43 ± 0.08 × 10^2^; *D*: 3.26 ± 0.66 mm^2^/min) and Kiyomi (*k*: 0.83 ± 0.03 × 10^2^; *D*: 1.59 ± 0.15 mm^2^/min) (*p* < 0.05) (Figure 2D,E).

Moreover, scanning electron microscopy (SEM) of the peel morphology of the three citrus varieties showed that the stoma size in Yellowball was more than 10 times and 2 times smaller than that in Haruka and Kiyomi, respectively (Figure 2F). Furthermore, the cellular structure of albedo tissue in Yellowball was more compact than that in Haruka and Kiyomi.

### 2.4. Metabolomics Analysis

**Figure 3:**
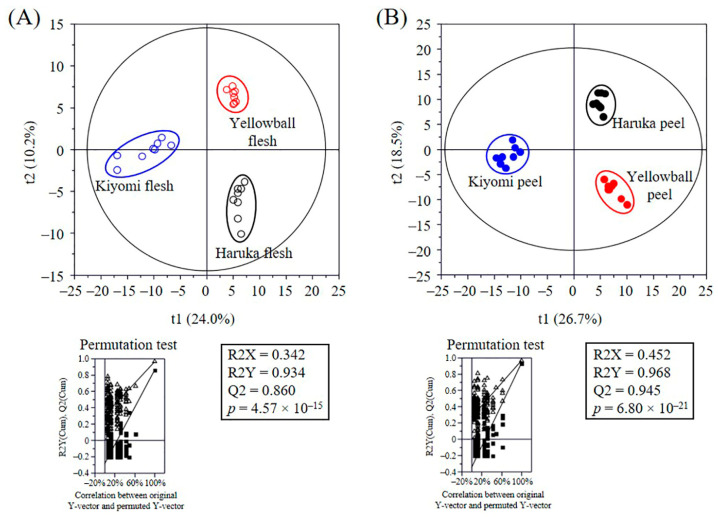
Partial least-squares discriminant analysis (PLS-DA) score plots of citrus metabolites in Haruka, Kiyomi, and Yellowball and their quality parameters. PLS-DA score plot of flesh (**A**) and peel (**B**) metabolites. Metabolites were analyzed using gas chromatography–mass spectrometry (GC-MS), ultra-performance liquid chromatography–quadrupole–time-of-flight MS (UPLC-Q-TOF MS), and high-performance liquid chromatography (HPLC). Statistical acceptance of the PLS-DA model was evaluated by calculating its R2X, R2Y, Q2, and *p*-value and validated using a permutation test and cross-validation (n = 200).

The flesh and peel metabolite profiles of the three citrus varieties were analyzed using gas chromatography–mass spectrometry (GC-MS), ultra-performance liquid chromatography–quadrupole–time-of-flight mass spectrometry (UPLC-Q-TOF-MS), and high-performance liquid chromatography (HPLC) (Figures S3–S6), and the statistical discrimination between these citrus varieties was visualized using partial least-squares discriminant analysis (PLS-DA score plots (Figure 3). The goodness of fit (R2X = 0.342 and 0.452; R2Y = 0.934 and 0.968), predictability (Q2 = 0.860 and 0.945), *p*-values (6.80 × 10^−21^ and 4.57 × 10^−15^), and cross-validation of the permutation test (Y-axis of R < 0.3 and Q < −0.3) showed that the PLS-DA models were statistically acceptable. The score plots showed that the three citrus varieties were clearly separated by t1 and t2.

To find metabolites contributing to the separation between samples in PLS-DA score plots, the variable importance in the projection (VIP) and *p*-values of all individual metabolites were calculated (Tables S2–S4). Sixty-nine metabolites with a VIP > 0.7 and *p*-value < 0.05 were identified, including five sugars, eight amino acids, ten acidic compounds, ten lipids, twenty-two flavonoids, two limonoids, eight carotenoids, and four other compounds.

### 2.5. Citrus Metabolomic Pathway and Relative Abundance of Identified Metabolites

**Figure 4:**
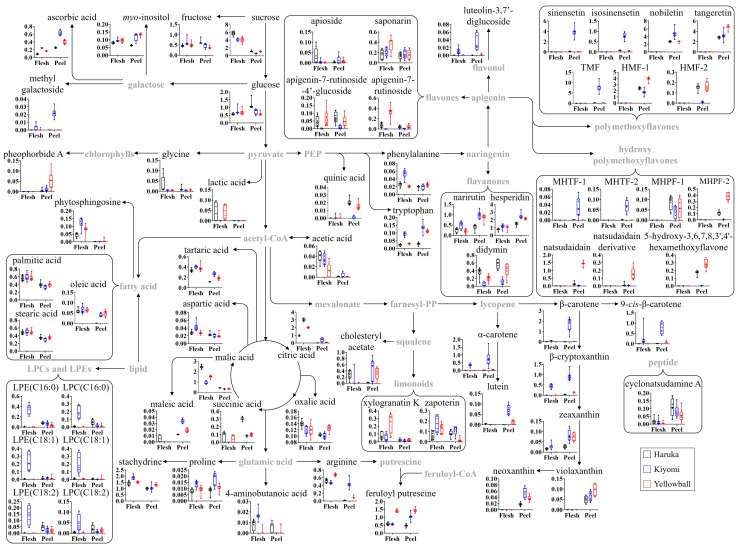
Schematic diagram of the metabolomic pathway and relative abundance of citrus metabolites in flesh and peels of Haruka, Kiyomi, and Yellowball. Y-axis shows the normalized chromatogram intensity and X-axis shows the citrus flesh and peel. White, blue, and red indicate Haruka, Kiyomi, and Yellowball, respectively. Organic acids were quantitatively analyzed using authentic standards. LPE, lysophosphatidylethanolamine; LPC, lysophosphatidylcholine; TMF, tetramethoxyfalvone; HMF, hexamethoxyflavone; MHTF, monohydroxy tetramethoxyflavone; MHPF, monohydroxy pentamethoxyflavone.

Based on the identified citrus metabolites, the citrus metabolomic pathway, including primary and secondary metabolites, was proposed, and the relative metabolite abundance was compared among the citrus varieties (Figure 4). The relative abundance of primary and secondary metabolites significantly differed among the three varieties. In primary metabolites, sugars and acidic compounds were the major primary metabolites in the three citrus varieties. Most sugars in the flesh and peels did not differ among the three varieties, except for sucrose in the flesh and glucose, *myo*-inositol, and methyl galactoside in the peels. Sucrose in Haruka flesh and glucose in Haruka peels were 1.4–1.9 times higher than those in the other varieties, while *myo*-inositol in Haruka peels was more than 1.8 times lower than that in the other varieties. Methyl galactoside was only observed in Kiyomi peels. Conversely, the levels of major acidic compounds, including citric, malic, tartaric, ascorbic, and succinic acid, significantly differed according to the variety. Citric acid levels in Kiyomi flesh were 3.1- and 1.5 times higher than that in Haruka and Yellowball flesh, respectively; ascorbic acid showed a similar pattern. However, malic acid levels in Kiyomi flesh were 2.7 and 1.7 times lower than those in Haruka and Yellowball flesh, respectively. Ascorbic, tartaric, and maleic acid levels in Kiyomi peels were 2.3–2.8 times and 1.5–1.8 times higher than those in Haruka and Yellowball peels, respectively, while succinic acid levels in Haruka peels were more than 3 times higher than that in other varieties.

In term of lipids, fatty acids were not significantly different among the three citrus varieties, except that oleic acid was absent only in Haruka peel. However, elevated lysophosphatidylethanolamine (LPE) and lysophosphatidylcholine (LPC) levels were observed only in Kiyomi flesh, in which the highest level of phytosphingosine was observed.

Regarding amino acids, Kiyomi had the highest levels of phenylalanine, tryptophan, and stachydrine in the flesh and arginine and tryptophan in the peel compared with those in the other varieties, while the highest level of arginine was observed in Yellowball flesh.

In addition to primary metabolites, the profiles of secondary metabolites, including flavones, flavanones, polymethoxyflavones (PMFs), hydroxyl polymethoxyflavones (HPMFs), carotenoids, and limonoids, were significantly different among the citrus varieties. Among these secondary metabolites, PMFs, HPMFs, and carotenoids were mainly observed in citrus peels only, except for some carotenoids in Kiyomi flesh (α-carotene, β-cryptoxanthin, 9-*cis*- β-carotene, and zeaxanthin). In particular, major carotenoids (α-carotene, β-carotene, 9-*cis*-β-carotene, and β-cryptoxanthin), sinensetin, isosinensetin, tetramethoxyflavone (TMF), and monohydroxy tetramethoxyflavones (MHTFs) were observed in Kiyomi peels only, while the highest levels of tangeretin, heptamethoxyflavone-1 (HMF-1), monohydroxy pentamethoxyflavone-2 (MHPF-2), natsudaidain, natsudaidain derivative, and 5-hydroxy-3,6,7,8,3′,4′-hexamethoxyflavones were observed in Yellowball peels. Regarding flavanones and flavones, didymin and apigenin glucosides (apigenin-7-rutinoside-4′-glucoside and apigenin-7-rutinoside) were mainly observed in Haruka and Yellowball peels and flesh, and their levels were more than 4.5 times higher than those in Kiyomi. Narirutin and hesperidin levels in Kiyomi and Yellowball peels were approximately 2 times higher than those in Haruka. Furthermore, a higher level of feruloyl putrescine was observed in the flesh and peel of Yellowball compared with that in Haruka and Kiyomi.

### 2.6. Antioxidant and Anti-Inflammatory Activities of Peel Extracts

**Figure 5:**
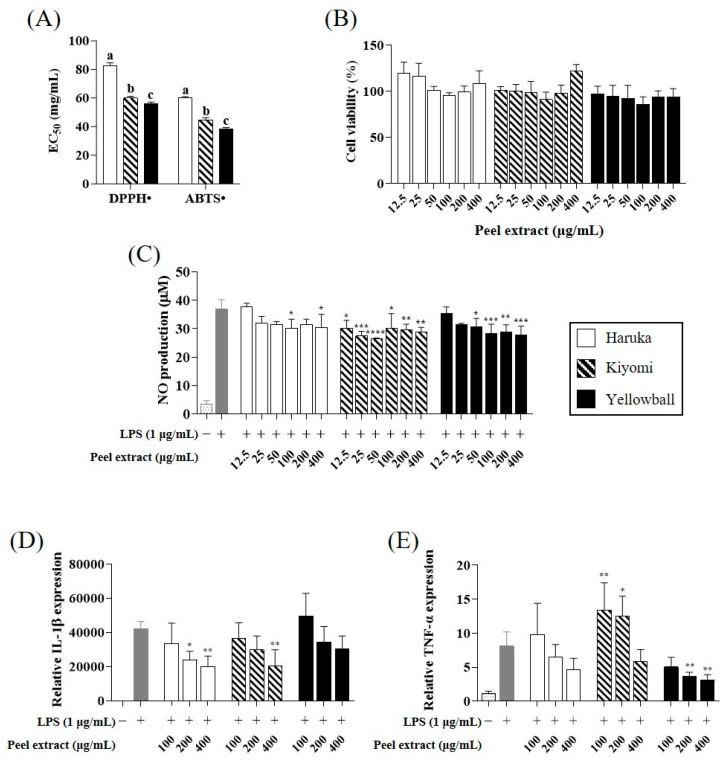
Antioxidant (**A**) and anti-inflammatory activities of Haruka, Kiyomi, and Yellowball peel extracts. Cell viability (**B**) was measured using the MTT assay after 24 h. Anti-inflammatory activity of extracts was evaluated by testing NO production (**C**) and expression levels of inflammation-related cytokines, including Il-1β (**D**) and TNF-α (**E**) in LPS-induced RAW 264.7 cells. Different letters in each bar indicate a significant difference as determined by Duncan’s test (*p* < 0.05). * *p* < 0.05; ** *p* < 0.01; *** *p* < 0.001; and **** *p* < 0.0001 compared to LPS treatment using one-way ANOVA with Dunnett’s test. −, non-treatment; +, LPS treatment.

Analysis of the antioxidant and anti-inflammatory activities of each peel extract (Figure 5) revealed that Yellowball peel extract had the lowest half-maximal effective concentration (EC50) values for 2,2-diphenyl-1-picrylhydrazyl (DPPH•, 56.1 mg/mL) and 2,2′-azino-bis(3-ethylbenzothiazoline-6-sulfonic acid) (ABTS•, 38.6 mg/mL) compared with those of Haruka (DPPH• = 82.5 mg/mL; ABTS• = 60.4 mg/mL) and Kiyomi (DPPH• = 60.0 mg/mL; ABTS• = 44.9 mg/mL) (*p* < 0.05).

The anti-inflammatory activity of the peel extracts was evaluated in lipopolysaccharide (LPS)-induced RAW-264.7 cells in terms of nitric oxide (NO) production and mRNA expression of pro-inflammatory cytokines, including interleukin-1β (IL-1β) and tumor necrosis factor-α (TNF-α). LPS-induced NO production was reduced by the addition of all three peel extracts, but there was no significant difference between samples (Figure 5C). Conversely, Haruka and Kiyomi peel extracts suppressed IL-1β expression at increasing concentrations from 100–400 μg/mL (*p* < 0.05) (Figure 5D). In contrast, Yellowball peel extract had a significant effect on TNF-α suppression, which was decreased by up to 61% at a concentration of 400 μg/mL, compared with that of the control (*p* < 0.05) (Figure 5E).

### 2.7. Correlation Analysis

**Figure 6:**
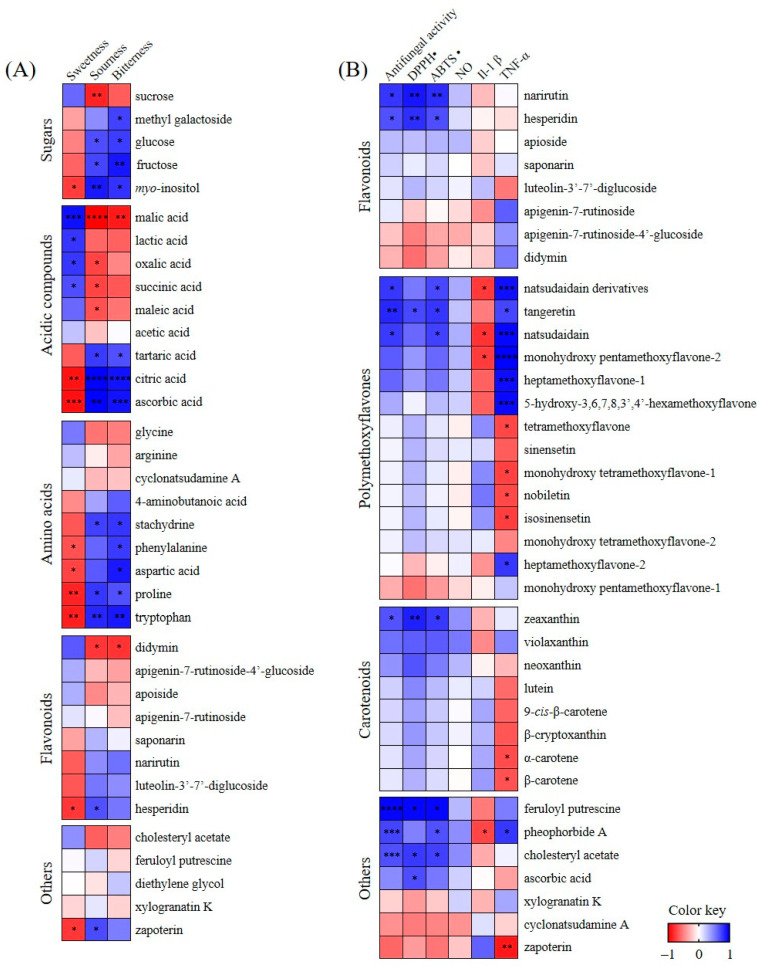
Pearson’s correlation coefficient between flesh metabolites and sensory analysis (**A**) or peel metabolites, antifungal activity, and functional properties (**B**). Colors of the correlation heat map represent the correlation coefficients; blue and red on a red–blue color scale indicate positive and negative correlations, respectively. SSC, soluble solids content; TA, titratable acidity; DPPH, DPPH radical scavenging activity; ABTS, ABTS radical scavenging activity. NO, Il-1β, and TNF-α indicate suppression of LPS-induced nitric oxide, Il-1β, and TNF-α production in RAW 264.7 cells, respectively. * *p* < 0.05; ** *p* < 0.01; *** *p* < 0.001; **** *p* < 0.0001.

The Pearson correlation coefficients between metabolites, sensory analysis results, and functional properties were analyzed and visualized using heatmaps (Figure 6). In the correlation between flesh metabolites and sensory analysis results (Figure 6A), sourness and bitterness had positive correlations (*r* > 0.78) with citric acid, ascorbic acid, tryptophan, and *myo*-inositol, and a negative correlation with malic acid (*r* < −0.88). However, sweetness had a negative correlation with sourness and bitterness.

In the correlation between peel metabolites and antifungal activity (Figure 6B), narirutin, hesperidin, tangeretin, natsudaidain compounds, zeaxanthin, feruloyl putrescine, pheophorbide A, and cholesteryl acetate had positive correlations with antifungal and antioxidant activities (*r* > 0.67). Feruloyl putrescine had a strong positive correlation with antifungal activity (*r* > 0.95). Natsudaidain compounds, monohydroxy pentamethoxyflavone-2, heptamethoxyflavone-1, and 5-hydroxy-3,6,7,8,3′,4′-hexamethoxyflavone had strong positive correlations with TNF-α expression levels (*r* > 0.91).

## 3. Discussion

Numerous citrus varieties are being newly developed to improve citrus quality globally [11,12,13,16,17,18]. However, the quality of many of these varieties has not been objectively evaluated. The quality characteristics of Yellowball, a recently developed citrus resulting from the hybridization of Haruka and Kiyomi, have not been evaluated and compared with those of other citrus varieties. Thus, we evaluated the quality characteristics of Yellowball in terms of sensory quality, storability, metabolite profiles, and functionalities, and compared them with those of Haruka and Kiyomi.

Among various quality characteristics, the storage properties differed greatly among the three citrus varieties. The appearance of Yellowball hardly changed during the 60-day storage period, while Haruka and Kiyomi decayed. Considering that the storage period of other citrus varieties, including Haruka and Kiyomi, is generally below one month [19], Yellowball showed excellent storability because of the strong antifungal activity and dense structural characteristics of its peel (Figure 2B,F). In the antifungal test against *P. digitatum*, a major pathogen of postharvest decay in citrus fruits [20], the Yellowball peel extract showed stronger antifungal activity than its parent varieties. Citrus flavonoids, including naringin, hesperidin, PMFs (tangeretin, HMF, nobiletin, and sinensetin), and HPMFs are known antifungal agents against *P. digitatum* [21] and might contribute to the antifungal activities of Yellowball and its parent varieties. Among these compounds, tangeretin, HMFs, and HPMFs, which were relatively abundant in Yellowball peels, as well as feruloyl putrescine, which has strong antifungal activity against some plant pathogens including *Aspergillus niger*, *Fusarium culmorum*, and *P. verucosum* [22], were considered to be specifically involved in the antifungal activity of Yellowball.

The structural characteristics of the peel were also strongly involved in citrus storability. In general, citrus peels have a high stomatal density (around 1400/cm^2^) and a fast water vapor permeability [23]; thus, a thicker peel improves its storability [24]. However, although the Yellowball had a relatively thin peel, its WVP was slower than those of its parent varieties (Figure 2C–E) because the stomata size was relatively small and the albedo layer was condensed (Figure 2F). The condensed structure of the albedo layer in mandarin peels also contributes to the reduction in gas diffusion compared with that of grapefruit peels [25]. In lemons, the chitosan-based coating in their peels reduces the number of opened stoma and contributes to transpiration inhibition and senescence delay [26]. Furthermore, the reduced size and number of stoma in lemon peels inhibits pathogen infection because the stoma is the main pathogen invasion site [26].

In addition to the storage characteristics, citrus quality was considerably different between Yellowball and its parent varieties. The citrus metabolite profile, a crucial factor associated with the physicochemical and sensory qualities of citrus [7,27], was significantly different among the varieties (Figure 4). Carotenoid profiles are strongly correlated with citrus color [13]. High β-carotene and β-cryptoxanthin levels contributed to the orange or red color of Kiyomi, while the yellow color of Yellowball and Haruka was due to their relatively low carotenoid levels.

The citrus taste is a result of a combination of various metabolites involved in basic taste (sugars, acidic compounds, phenolic compounds, and limonoids), aroma (volatile compounds), and mouth-feel sensations (pectins, cellulose, and hemicellulose) [28]. In particular, the balance of sweetness and sourness evaluated by SSC, TA, and the SSC/TA ratio is mainly recognized as the dominant citrus taste [29]. In this study, the dominant sourness-related organic acids, such as citric, malic, and ascorbic acid, contributed more to the taste of the three citrus varieties than sweet sugars, including sucrose, fructose, and glucose. These organic acids influence the overall taste of citrus fruits by stimulating bitterness and suppressing or partially masking sweetness [29,30]. Feruloyl putrescine enhances perceived sweetness because of reduced bitterness [2]. Despite the similar sugar contents between Yellowball and Kiyomi, Yellowball was sweeter than Kiyomi because of organic acids, as well as feruloyl putrescine, which was abundant in Yellowball flesh.

In addition to taste quality, citrus secondary metabolites also play a key role in the various health benefits of citrus, including their antioxidant, anti-inflammatory, anticancer, antitumor, antigenotoxic, anti-obesity, and antidiabetic activities [3,4]. In this study, the profile difference in major flavonoids, including hesperidin, narirutin, tangeretin, HPMFs, and PMFs, which are known antioxidants [31,32] or anti-inflammatory compounds [33], contributed to the difference between the antioxidant and anti-inflammatory activities of the citrus peel extracts (Figure 5A). Previous reports show that natsudaidain and 5-hydroxy-3,6,7,8,3′,4′-hexamethoxyflavone, which were highly abundant in Yellowball peels (Figure 4), strongly suppressed TNF-α expression in RBL-2H3 and RAW 264.7 cells, respectively [34,35], similar to our results.

## 4. Materials and Methods

### 4.1. Sample Preparation

Yellowball, Haruka, and Kiyomi harvested in March 2022 were obtained from the Citrus Research Institute in Jeju, Republic of Korea. The flesh and peels of each citrus variety were separated. Some of the fresh flesh portions were immediately used to measure sensory quality, SSC, and TA, and the some of the fresh peels were immediately used to measure water vapor permeability. The remaining portions of flesh and peels were freeze-dried and stored at −80 °C until use.

### 4.2. Chemicals

Food-grade sucrose and citric acid were purchased from a local market and the ES Ingredients Co., Ltd. (Gunpo, Republic of Korea). The solvents, including methanol, ethanol, acetonitrile, hexane, and methyl tert-butyl ether (MTBE), were purchased from J.T. Baker (Phillipsburg, NJ, USA), and dimethyl sulfoxide (DMSO) was purchased from GenDEPOT (Baker, CA, USA). Potato dextrose agar (PDA), Dulbecco’s modified Eagle’s medium (DMEM), fetal bovine serum (FBS), and penicillin/streptomycin were purchased from Gibco (Grand Island, NY, USA), and TRIzol reagent was purchased from Invitrogen Co. (Waltham, ME, USA). The remaining chemicals were purchased from Sigma-Aldrich (Saint Louis, MO, USA).

### 4.3. Color, SSC, TA

The color values, including lightness (L*), redness (a*), and yellowness (b*), were measured using a hand-held colorimeter (CR-400; Minolta, Tokyo, Japan), and the CCI of flesh was calculated using the following equation [7]:

(1) $$\mathrm{CCI}=\frac{\left(1000\times a*\right)}{\left(b*\times L*\right)}$$

The fresh flesh juice was centrifuged at 10,000× *g* at 4 °C for 15 min, and the SSC (%) of the supernatant was measured using a handheld reflectometer (Daihan Scientific Co., Wonju, Republic of Korea). The TA (% citric acid in fresh weight) of the supernatant was measured by mixing 1 mL of supernatant, 5 mL of distilled water, and 0.2 mL of 1% phenolphthalein and titrating it with 0.01 N NaOH solution to the endpoint. SSC and TA were measured using three replicates.

### 4.4. Sensory Evaluation

Sensory evaluation was performed by nine trained panelists, aged 21–32 years, and approval was obtained from the Human Research Ethics Committee of Gyeongsang National University (Approval Number GIRB-G22-Y-0063). All panelists were trained in sensory evaluation for more than three months. Before sensory evaluation, all panelists discussed a series of taste reference solutions, including sucrose (8%) for sweetness, citric acid (0.2%) for sourness, and quinine (0.0025%). Purified water was provided as a palate cleanser. The intensity of each sensory quality was rated on a 15 cm line scale labeled “very weak” and “very strong” with 0.5 cm anchors on the left and right sides [36].

### 4.5. WVP

The WVP of the peels was measured using the Salazar method [37] with some modifications. The peels were fixed with a polytetrafluoroethylene band to the screw-cap of a glass bottle containing 10 g of anhydrous ammonium sulfate (AAS), after which the bottle was completely closed and placed in a desiccator with distilled water that maintained 100% of the internal relative humidity. The moisture weight (g) absorbed in the AAS was measured every 12 h for 96 h. The WVP rate constant was calculated using a first-order kinetic model with the following equation:

(2) $$\frac{W_{t}}{W_{0}}=1-\exp(-kt)$$

where *W_t_* is the mass (g) of the water with the peel absorbed in AAS at time (*t*), *W*_0_ is the mass of the water without the peel, and *k* is the rate constant.

The diffusion coefficient (*D*, mm^2^/min) of water vapor in the peel was calculated using Fick’s second law of diffusion with the following equation [38]:

(3) $$\frac{W_{t}}{W_{0}}=1-(\frac{8}{\pi^{2}})\exp(\frac{-\pi^{2}Dt}{l^{2}})$$

where *l* is the peel thickness (Table S1). The *ln*(1 − *W_t_*/*W*_0_) versus time (*t*) plot was used to calculate a linear line, and the diffusion coefficient (*D*) was calculated from the slope and peel thickness (*l*) using the following equation:

(4) $$D=\mathrm{Slope}(\frac{l^{2}}{\pi^{2}})$$

### 4.6. Scanning Electron Microscopy

The freeze-dried peel samples were coated with 10 nm pure platinum using an ion coater (KIC-1A, Coxem, Daejeon, Republic of Korea) and their morphology was examined using a scanning electron microscope (Apreo S; ThermoFisher Scientific, Waltham, MA, USA) at 50–1600× magnification using an accelerating voltage of 15 kV.

### 4.7. Antifungal, Antioxidant, and Anti-Inflammatory Activity

#### 4.7.1. Sample Preparation

Lyophilized citrus peel powder (50 g) was extracted with 500 mL of 70% (*v*/*v*) aqueous methanol for 30 min under sonication. After centrifugation (3000× *g* for 30 min at 25 °C), the supernatant was collected, and the residues were re-extracted twice with 500 mL of 70% aqueous methanol. The collected supernatants were concentrated and freeze-dried, and then re-dissolved in DMSO.

#### 4.7.2. Antifungal Activity

The antifungal activity of peel extracts against citrus green mold (*P. digitatum*) was determined using a modified protocol described by Ruiz et al. [39]. *P. digitatum* was isolated from naturally decaying Haruka and cultured on potato dextrose (PD) agar at 25 °C. The stock solution of spore suspension was adjusted to 1 × 10^8^ spores/mL. Peel extracts with different concentrations (2.5–80 mg/mL) were mixed with the spore suspension to achieve a spore concentration of 1 × 10^6^ spores/mL. The prepared mixtures were incubated at 25 °C for 12 h in the dark. Then, 5 μL of each mixture was spotted onto the center of Petri dishes prepared with PD agar containing chloramphenicol (850 μg/mL) as an antibiotic. Colony diameters were measured after incubation for 7 d at 25 °C.

#### 4.7.3. Antioxidant Activity—DPPH and ABTS Assays

The antioxidant activity of the peel extracts was measured using the radical scavenging activities against DPPH• and ABTS•. The DPPH radical scavenging assay was performed using the Blois method [40] with a minor modification. The reaction mixture of the diluted peel extracts (10 μL) and 0.2 mM DPPH solution (200 μL) were incubated at 25 °C in the dark for 30 min; absorbance was measured at 520 nm. For the ABTS radical cation scavenging assay [41], 10 μL of diluted peel extract was mixed with 200 μL of ABTS radical cation solution (0.2 mM) and incubated at room temperature in the dark for 8 min; absorbance was measured at 734 nm. The antioxidant activities of the peel extracts were expressed as the EC50 (mg/mL).

#### 4.7.4. Anti-Inflammatory Activity

The viability of RAW 264.7 cells in different peel extract concentrations was evaluated using the MTT method. The cells were grown in DMEM with 10% FBS and 1% penicillin (100 U/mL)/streptomycin (100 μg/mL) under a humidified condition of 5% CO_2_ at 37 °C. Cells (1 × 10^5^ cells/well) were pretreated with various peel extract concentrations (12.5–400 μg/mL) for 48 h and then starved in DMEM for 12–18 h before LPS stimulation (1 μg/mL) for 24 h in 1% FBS-containing medium with or without peel extracts. NO production was measured using Griess reagent, and the gene expression of IL-1β and TNF-α was measured through real-time polymerase chain reaction (PCR). RNA was extracted using TRIzol reagent and quantified using a Nano-200 Micro-Spectrophotometer (NanoDrop, Hangzhou City, China), and gene expression was determined through real-time PCR (CFX96™ Real-Time PCR Detection System; Bio-Rad, Hercules, CA, USA). The specific forward and reverse primer sequences were as follows: IL-1β (Forward: 5′-AAATACCTGTGGCCTTGGGC-3′, Reverse: 5′-CTTGGGATCCACACTCTCCAG-3′); TNF-α (Forward: 5′-GGCTGCCCCGACTACGT-3′, Reverse: 5′-ACTTTCTCCTGGTATGAGATAGCAAAT-3′). Relative gene expression was normalized to that of ribosomal protein lateral stalk subunit P0 or hypoxanthine–guanine phosphoribosyltransferase [42].

### 4.8. Citrus Metabolomics Analysis

#### 4.8.1. GC-MS Analysis

The lyophilized flesh and peel samples were homogenized with 70% aqueous methanol containing dicyclohexyl phthalate as an internal standard (IS). After centrifugation, the supernatant was completely dried using a centrifugal vacuum concentrator (CentriVap^®^, Labconco Co., Kansas City, MO, USA). The dried residues were re-dissolved in 70 μL of methoxyamine hydrochloride in pyridine (20 mg/mL) and incubated at 37 °C for 90 min. The samples were derivatized by adding 70 μL of N,O-bis(trimethylsilyl)trifluoroacetamide with 1% trimethylchlorosilane at 70 °C for 30 min. Next, 1 μL of the derivatized sample was injected into the GC-2010 plus system (Shimadzu Corp., Kyoto, Japan) equipped with a DB-5 ms capillary column (30 m × 0.25 mm, 0.25 μm, Agilent J&W column; Agilent Technologies, Santa Clara, CA, USA) with a split ratio of 1:20. The injector temperature was 200 °C. Helium was used as a carrier gas with a gas flow rate of 1 mL/min. The oven temperature was maintained at 70 °C for 2 min, increased to 150 °C at 5 °C/min, 210 °C at 3 °C/min, 320 °C at 8 °C/min, and finally held at 320 °C for 8 min. The eluents were detected using a GCMS-TQ 8030 MS system (Shimadzu Corp., Kyoto, Japan) with electron ionization at 70 eV. Ion source and interface temperatures were set to 200 °C and 250 °C, respectively. The data were monitored and collected in full-scan mode in the mass range of m/z 45–550 with a scan event time of 0.3 s and a scan rate of 2000 amu/s. The quality control sample, which was prepared by mixing all samples, was analyzed once between each sample set [7].

#### 4.8.2. UPLC-Q-TOF MS Analysis

The lyophilized flesh and peel samples were homogenized with 70% aqueous methanol containing terfenadine as an IS to extract metabolites. After centrifugation, the supernatants were analyzed using an UPLC-Q-TOF-MS (XevoTM G2-S; Waters Corp., Milford, MA, USA) equipped with an Acquity UPLC BEH C18 column (2.1 × 100 mm, 1.7 μm, Waters Corp.). The column was equilibrated with water containing 0.1% formic acid (FA) (solvent A) at 40 °C with a flow rate of 0.35 mL/min, and the metabolites were eluted with a gradient of acetonitrile containing 0.1% FA (solvent B) as follows: from 0 to 1 min, 0% B; from 1 to 8 min, a linear gradient from 0 to 100% B; from 8 to 9 min, 100% B, from 9 to 10 min, 0% B; from 10 to 12 min, 0% B. The eluted metabolites were detected using a Q-TOF-MS with a positive electrospray ionization (ESI) mode under optimized MS conditions (desolvation gas flow rate, 800 L/h; desolvation temperature, 400 °C; ion source temperature, 100 °C; capillary voltage, 3 kV; sampling cone voltage, 40 V). Leucine-enkephalin ([M + H] = 556.2771) was used as a lock mass reference, and the TOF-MS scan range was m/z 100–1500. The MS/MS spectra were obtained using a collision energy ramp from 10–30 eV or 20–40 eV [7].

#### 4.8.3. HPLC Analysis of Organic Acid, Ascorbic Acid, and Carotenoids

To analyze organic and ascorbic acids, the lyophilized flesh and peel samples were homogenized with 5% aqueous meta-phosphoric acid. After centrifugation, the supernatants were analyzed using a HPLC (Shimadzu Corp.) with a photodiode array (PDA) detector (Shimadzu Corp.). The extracted samples were injected into a Triart C18 column (250 × 4.6 mm, 4.6 mm I.D., 5 μm; YMC Co., Ltd., Kyoto, Japan.) and eluted with water containing 0.1% phosphoric acid. A flow rate of 0.35 mL/min and column temperature of 40 °C were employed. The eluted compounds were detected at 220 nm for organic acids and 254 nm for ascorbic acid. The organic and ascorbic acids were identified by comparing their retention times with authentic standards [7].

For carotenoid analysis, the lyophilized flesh and peel samples (0.1 g) were extracted using 5 mL of ethanol–hexane (4:3, *v*/*v*) with Na_2_CO_3_ (0.25 g) and 2,6-di-tert-butyl-methylphenol (BHT; 0.05 g) by shaking at room temperature for 1 h. After centrifugation, the supernatant was recovered, and the residue was re-extracted twice with 5 mL of hexane. The combined supernatant was washed with 20 mL of distilled water and 20 mL of 10% NaCl and completely dried using a centrifugal vacuum concentrator (Labconco Co.). The dried residue was dissolved in MTBE and analyzed using a HPLC (Shimadzu Corp.) at 450 nm. The extracted samples were injected into a C30 column (YMC carotenoid C30, 250 × 4.6 mm I.D., 5 μm; YMC Co. Ltd., Kyoto, Japan) with a column temperature of 40 °C. The carotenoids were eluted with solvent A [methanol:MTBE:water (81:15:4)] and solvent B (MTBE) with gradient elution of solvent B from 0–66.6% for 60 min at a flow rate of 1 mL/min. The carotenoids were identified by comparing their retention times with authentic standards [7].

#### 4.8.4. Data Processing

The chromatographic responses obtained through GC-MS were aligned based on their retention times, normalized to the IS, and identified using NIST 11 and Wiley 9 mass spectral libraries, the retention indices (RI) of n-alkanes, and authentic standards. The MS data obtained through UPLC-Q-TOF MS were collected, aligned, and normalized using the MarkerLynx software (Waters Corp.). The metabolites were tentatively identified using the online database connected to the UNIFI software version 1.9.2 (Waters Corp.) and METLIN database [Scripps Center for Metabolomic, www.metlin.scripps.edu (accessed on 12 October 2022)].

### 4.9. Statistical Analysis

Multivariate statistical analysis was performed using SIMCA-P+ v.16.0.1 (Umetrics, Umeå, Sweden), and a PLS-DA score plot was used to visualize differences between sample groups. The differences between the experimental data were compared using one-way analysis of variance (ANOVA) with Duncan’s test (*p* < 0.05) using SPSS v.27.0 (SPSS Inc., Chicago, IL, USA). Pearson correlation coefficients between metabolites, sensory qualities, and functional properties were calculated and visualized through heatmaps created using GraphPad Prism 9.0 (GraphPad, San Diego, CA, USA).

## 5. Conclusions

The quality characteristics of a novel citrus variety, Yellowball, were evaluated in terms of appearance, storability, sensory properties, functionality, and metabolite profiles and compared with those of its parent varieties, Haruka and Kiyomi. The primary and secondary metabolite profiles differed significantly among the three citrus varieties, resulting in distinct physicochemical characteristics, including color, sensory quality, and antioxidant and anti-inflammatory activities. Moreover, the storability of Yellowball was significantly increased compared with that of its parent varieties due to its strong antifungal activity and unique peel morphology (stoma and albedo layer), despite its relatively thin peel. Furthermore, we postulated the citrus metabolomic pathways of the three varieties based on the identified metabolites. Although we did not investigate the volatile compounds, overall functional activities, and detailed characteristics of each metabolite, our data provide valuable insights into the relationship among citrus metabolites, peel morphology, physicochemical properties, and storability. Furthermore, our results demonstrate the potential of Yellowball as a novel variety in the citrus industry and provide information on the development of novel citrus fruits with unique and desirable qualities.

## Data Availability

The article contains all the information required to support its conclusions.

## Supplementary Materials

The following supporting information can be downloaded at: https://www.mdpi.com/article/10.3390/plants12152863/s1, Figure S1: Appearance changes in Haruka, Kiyomi, and Yellowball during storage for 60 days at 25 °C; Figure S2: Growth inhibition test of peel extracts on citrus green mold (*Penicillium digitatum*) at different concentration (2.5–80 mg/mL); Figure S3: Representative chromatograms of citrus metabolites analyzed by GC-MS; Figure S4: Representative chromatograms of citrus metabolites analyzed by UPLC-Q-TOF MS; Figure S5: Representative chromatograms of organic acids analyzed by HPLC at 220 nm; Figure S6: Representative chromatograms of carotenoids analyzed by HPLC at 450 nm; Table S1: Weight, width, length, and peel thickness of Haruka, Kiyomi, and Yellowball; Table S2: Identification of major metabolites by GC-MS; Table S3: Identification of major metabolites by UPLC-Q-TOF MS; Table S4: Identification of major metabolites by HPLC.
