# Supplementary material for: Comparative Analysis of Physicochemical Properties and Storability of a New Citrus Variety, Yellowball, and Its Parent
Source: Plants (Basel). 2023 Aug 3;12(15):2863. doi: 10.3390/plants12152863 (PMC10421519; doi:10.3390/plants12152863)
Supplement: Supplementary file 1 [file plants-12-02863-s001.zip › plants-2524260-supplementary.pdf]

# Supplementary Materials

**Table S1.** Weight, width, length, and peel thickness of Haruka, Kiyomi, and Yellowball.

| | Weight (g) | Width (cm) | Length (cm) | Peel thickness (cm) |
|---|---|---|---|---|
| Haruka | 331.49 ± 29.46 | 10.63 ± 0.38 | 10.09 ± 0.42 | 0.90 ± 0.09 |
| Kiyomi | 250.69 ± 18.83 | 9.39 ± 0.39 | 8.92 ± 0.37 | 0.68 ± 0.12 |
| Yellowball | 273.74 ± 15.47 | 9.76 ± 0.60 | 9.32 ± 0.37 | 0.44 ± 0.03 |

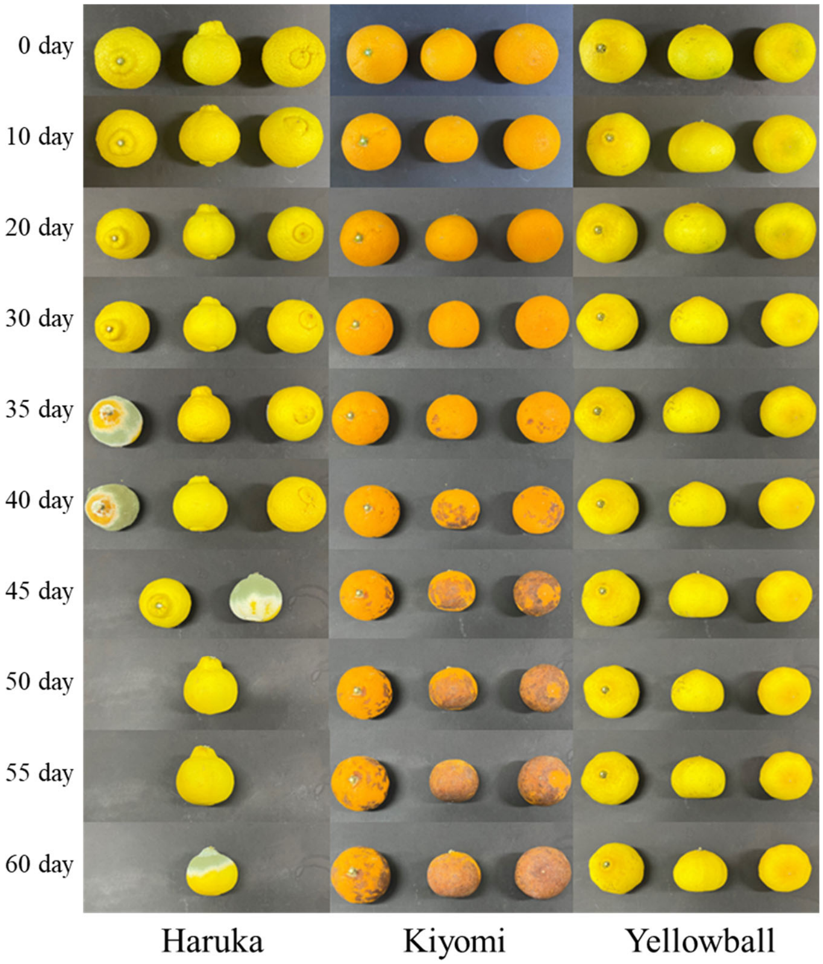


**Figure S1.** Appearance changes in Haruka, Kiyomi, and Yellowball during storage for 60 days at 25 °C.

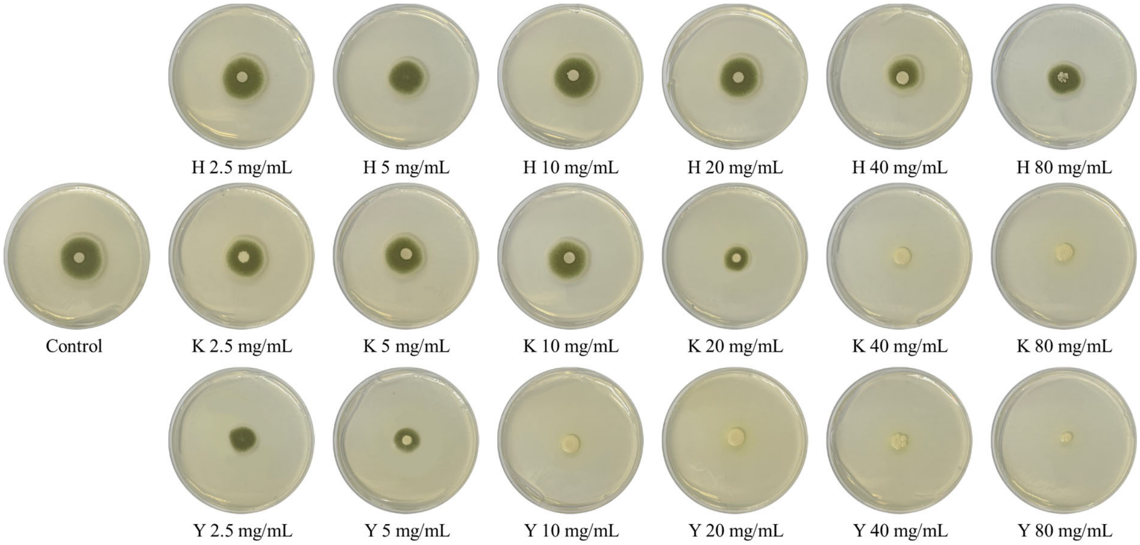


**Figure S2.** Growth inhibition test of peel extracts on citrus green mold (*Penicillium digitatum*) at different concentration (2.5–80 mg/mL). H, haruka; K, Kiyomi; Y, Yellowball.

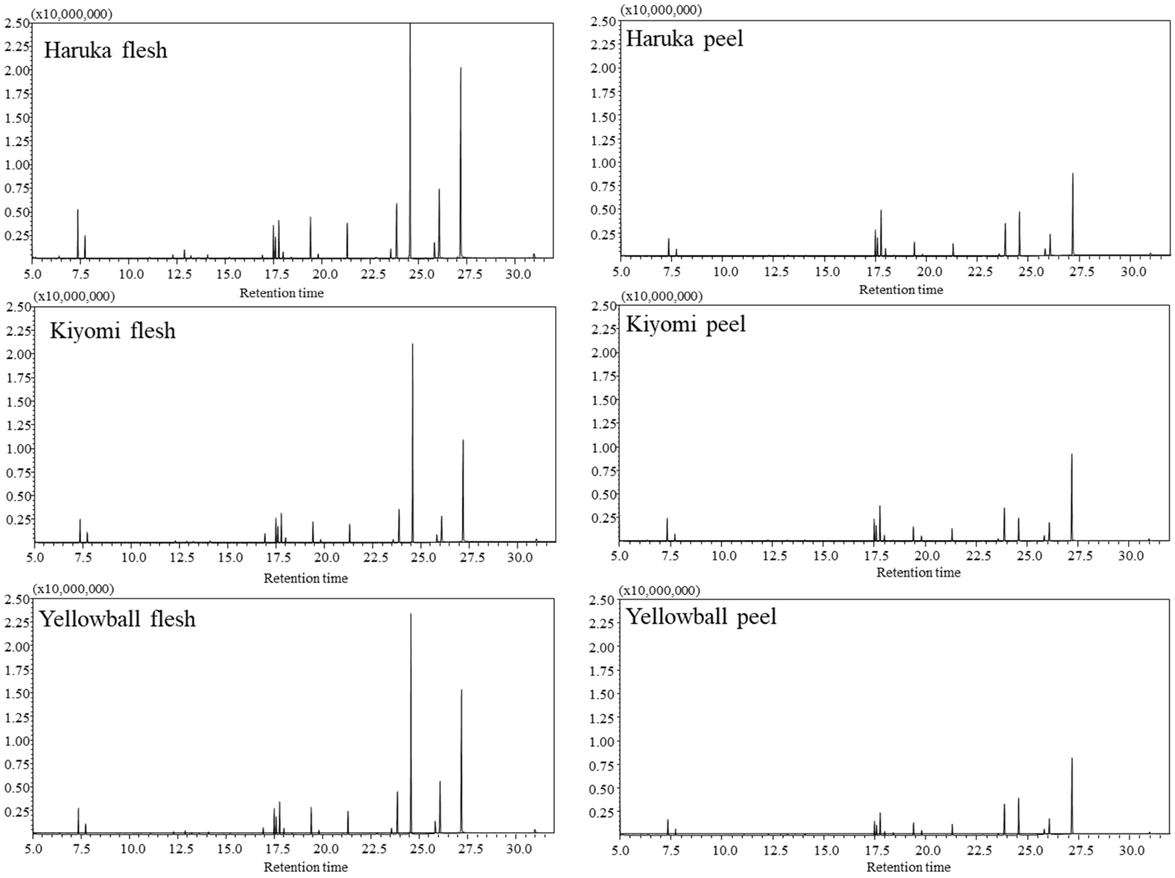


**Figure S3.** Representative chromatograms of citrus metabolites analyzed by GC-MS.

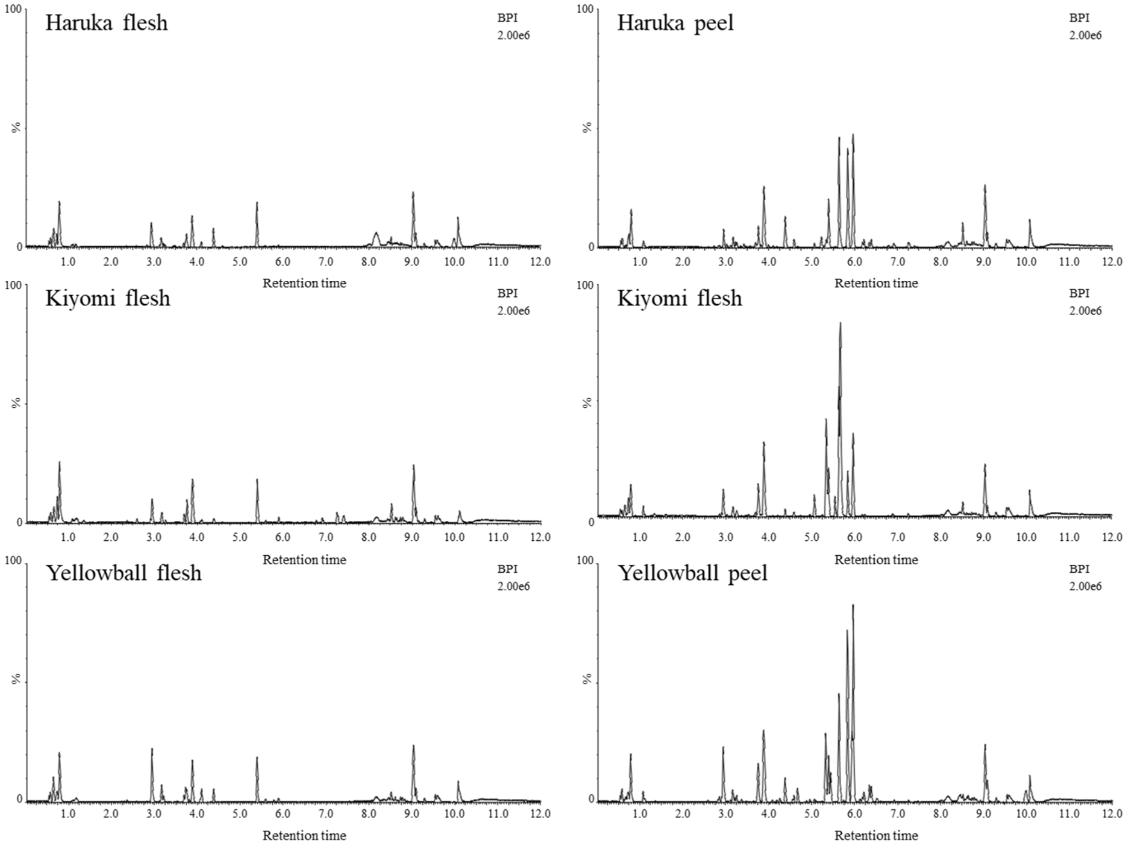


**Figure S4.** Representative chromatograms of citrus metabolites analyzed by UPLC-Q-TOF MS.

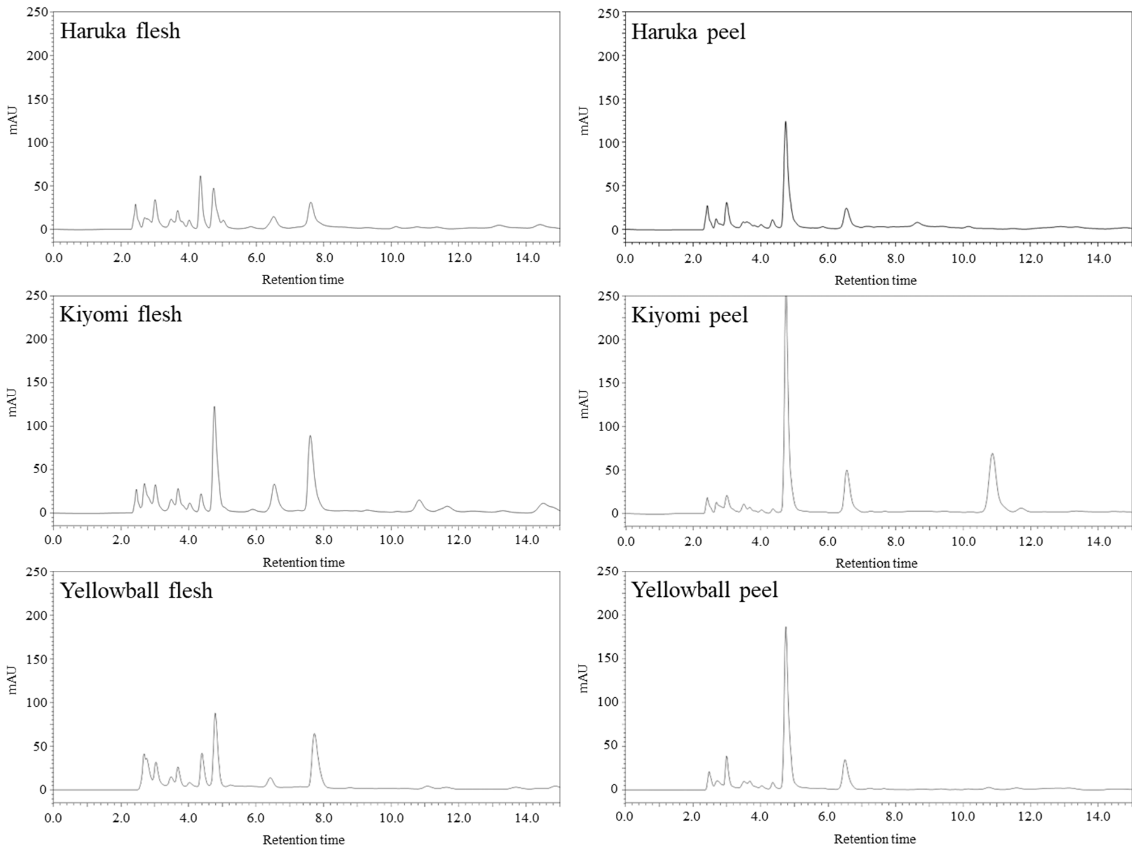


**Figure S5.** Representative chromatograms of organic acids analyzed by HPLC at 220 nm.

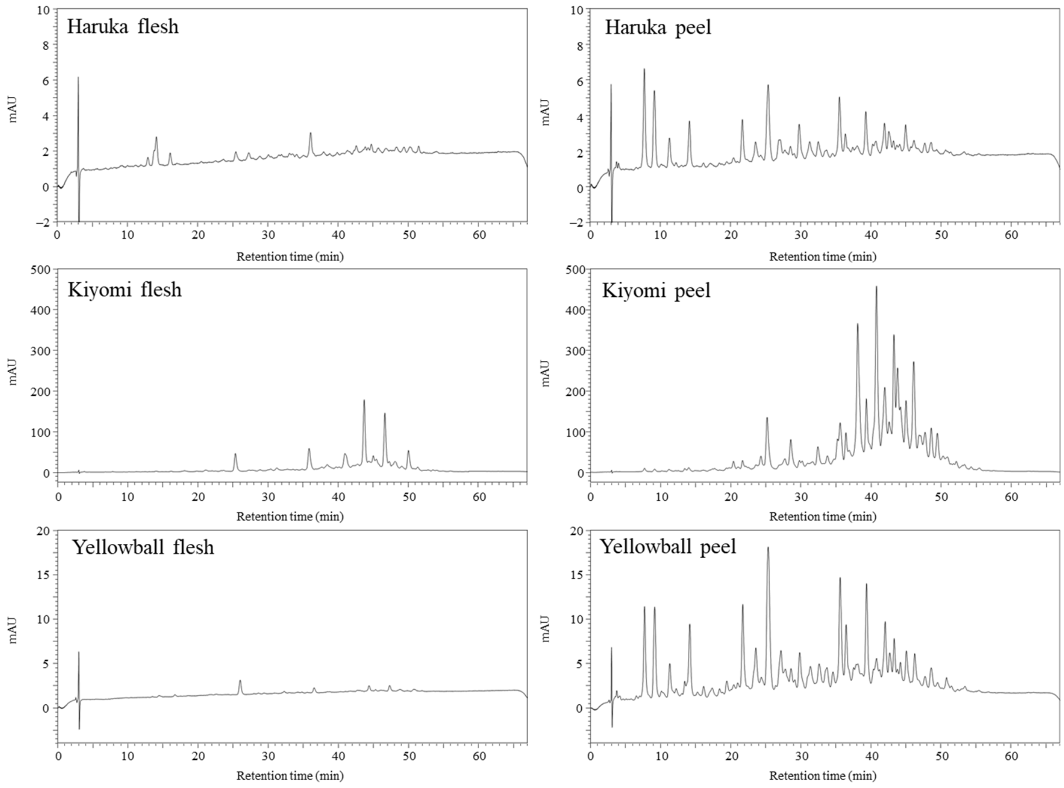


**Figure S6.** Representative chromatograms of carotenoids analyzed by HPLC at 450 nm.

**Table S2.** Identification of major metabolites by GC-MS.

| RT | Compounds | RI | VIP | *p*-value |
|---|---|---|---|---|
| 6.34 | glycine | 1051 | 1.21 | $2.45 \times 10^{-2}$ |
| 10.22 | proline | 1293 | 0.94 | $1.54 \times 10^{-6}$ |
| 13.48 | 4-aminobutanoic acid | 1525 | 1.32 | $1.10 \times 10^{-10}$ |
| 15.21 | aspartic acid | 1663 | 1.04 | $1.29 \times 10^{-8}$ |
| 17.34 | quinic acid | 1846 | 1.24 | $7.17 \times 10^{-20}$ |
| 17.49 | fructose | 1860 | 1.16 | $3.90 \times 10^{-5}$ |
| 17.77 | glucose | 1885 | 1.35 | $7.10 \times 10^{-7}$ |
| 18.14 | methyl galactoside | 1920 | 1.06 | $2.17 \times 10^{-19}$ |
| 19.41 | palmitic acid | 2022 | 0.81 | $2.32 \times 10^{-10}$ |
| 19.80 | *myo*-inositol | 2042 | 1.25 | $8.03 \times 10^{-13}$ |
| 21.30 | stearic acid | 2240 | 0.84 | $6.09 \times 10^{-12}$ |
| 24.51 | oleic acid | 2615 | 1.31 | $1.09 \times 10^{-14}$ |
| 24.57 | sucrose | 2622 | 1.21 | $8.29 \times 10^{-30}$ |

RT, retention time; RI, retention indices; VIP, variable importance in the projection.
*p*-Values were analyzed by Duncan's test.

**Table S3.** Identification of major metabolites by UPLC-Q-TOF MS.

| RT | Compounds | Exact mass (M+H) | MS fragment | VIP | *p*-value |
|---|---|---|---|---|---|
| 0.64 | arginine | 175.1181 | 70, 158, 116 | 1.27 | $6.60 \times 10^{-28}$ |
| 0.78 | stachydrine | 144.1010 | 116, 184, 70, 102, 58 | 1.15 | $6.02 \times 10^{-15}$ |
| 2.59 | phenylalanine | 166.0847 | 120, 103 | 1.39 | $2.22 \times 10^{-14}$ |
| 2.90 | tryptophan | 205.0959 | 188, 188, 144, 170 | 1.19 | $3.98 \times 10^{-22}$ |
| 2.93 | feruloyl putrescine | 265.1533 | 177, 145 | 1.81 | $2.19 \times 10^{-23}$ |
| 3.03 | luteolin-3'-7'-diglucoside | 611.1608 | 163, 593, 575, 325 | 0.90 | $1.41 \times 10^{-8}$ |
| 3.16 | saponarin | 595.1664 | 577, 475 | 1.33 | $1.91 \times 10^{-4}$ |
| 3.21 | apigenin-7-rutinoside-4′-glucoside | 741.2250 | 595, 433, 271, 377, 653 | 0.96 | $3.15 \times 10^{-4}$ |
| 3.48 | apioside | 565.1550 | 433, 415, 313, 521 | 1.31 | $2.36 \times 10^{-2}$ |
| 3.68 | zapoterin | 471.2016 | 425, 95 | 1.19 | $1.55 \times 10^{-6}$ |
| 3.72 | apigenin-7-rutinoside | 579.1719 | 271, 433, 519 | 1.77 | $7.64 \times 10^{-15}$ |
| 3.75 | narirutin | 581.1875 | 273, 419, 119 | 0.96 | $1.19 \times 10^{-15}$ |
| 3.88 | hesperidin | 611.1973 | 303, 449, 177 | 0.93 | $4.86 \times 10^{-18}$ |
| 4.09 | xylogranatin K | 515.2287 | 496, 409 | 1.50 | $5.87 \times 10^{-10}$ |
| 4.37 | didymin | 595.2021 | 287, 433, 559 | 1.19 | $3.53 \times 10^{-12}$ |
| 4.58 | cyclonatsudamine A | 728.3989 | 615, 587, 502, 474 | 0.95 | $2.81 \times 10^{-7}$ |
| 4.67 | natsudaidain derivatives (natsudaidain 3-(4-O-3-hydroxy-3-methylglutaroylglucoside)) | 725.2313 | 419 | 1.05 | $2.97 \times 10^{-13}$ |
| 4.73 | monohydroxy tetramethoxyflavone | 359.1125 | 184 | 0.88 | $5.36 \times 10^{-8}$ |
| 5.06 | isosinensetin | 373.1276 | 343, 358 | 1.08 | $4.13 \times 10^{-28}$ |
| 5.33 | sinensetin | 373.1269 | 343, 358 | 1.09 | $4.13 \times 10^{-25}$ |
| 5.63 | nobiletin | 403.1379 | 373, 355 | 0.94 | $1.04 \times 10^{-25}$ |
| 5.66 | tetramethoxyflavone | 343.1163 | 313, 282, 299, 281 | 1.08 | $9.13 \times 10^{-23}$ |
| 5.83 | heptamethoxyflavone | 433.1489 | 403, 385, 418 | 0.97 | $4.90 \times 10^{-34}$ |
| 5.88 | monohydroxy tetramethoxyflavone | 359.1123 | 184, 326 | 1.03 | $6.07 \times 10^{-16}$ |
| 5.89 | phytosphingosine | 318.3003 | 184, 282 | 1.12 | $5.03 \times 10^{-16}$ |
| 5.93 | natsudaidain | 419.1335 | 184, 389, 371 | 1.17 | $5.29 \times 10^{-38}$ |
| 5.96 | tangeretin | 373.1275 | 343, 358 | 0.92 | $1.17 \times 10^{-29}$ |
| 6.17 | monohydroxy pentamethoxyflavone-1 | 389.1235 | 359 | 1.00 | $7.52 \times 10^{-9}$ |
| 6.21 | heptamethoxyflavone | 403.1398 | 184, 373, 355 | 1.13 | $1.96 \times 10^{-22}$ |
| 6.34 | monohydroxy pentamethoxyflavone-2 | 389.1237 | 359, 374, 356, 341 | 1.09 | $3.43 \times 10^{-29}$ |
| 6.38 | 5-hydroxy-3,6,7,8,3',4'-hexamethoxyflavone | 419.1335 | 184, 389, 371 | 1.08 | $6.06 \times 10^{-26}$ |
| 6.90 | LPE(C18:2) | 478.2943 | 155, 337, 98, 460 | 1.46 | $2.14 \times 10^{-10}$ |
| 6.92 | LPC(C18:2) | 520.3410 | 337, 478, 104 | 1.16 | $1.39 \times 10^{-4}$ |
| 7.24 | LPE(C16:0) | 454.2945 | 184, 104, 125 | 1.58 | $1.12 \times 10^{-18}$ |
| 7.27 | LPC(C16:0) | 496.3406 | 184, 104, 125 | 1.49 | $2.20 \times 10^{-12}$ |
| 7.39 | LPE(C18:1) | 480.3086 | 155, 337, 98, 460 | 1.40 | $4.30 \times 10^{-12}$ |
| 7.41 | LPC(C18:1) | 522.3572 | 339, 341, 313, 104, 504 | 1.28 | $2.99 \times 10^{-9}$ |
| 8.63 | pheophorbide A | 593.2772 | 184 | 0.89 | $2.61 \times 10^{-7}$ |
| 9.98 | cholesteryl acetate | 429.3728 | 165, 205, 164, 219 | 1.23 | $6.61 \times 10^{-5}$ |

RT, retention time; VIP, variable importance in the projection; LPE, lysophosphatidylethanolamine; LPC, lysophosphatidylcholine.

*p*-Values were analyzed by Duncan's test.

**Table S4.** Identification of major metabolites by HPLC.

| | RT | Compounds | $\lambda_{max}$ (nm) | VIP | *p*-value |
|---|---|---|---|---|---|
| Organic acid | 3.1 | oxalic acid | | 1.39 | $7.11 \times 10^{-7}$ |
| | 3.5 | tartaric acid | | 1.12 | $1.68 \times 10^{-21}$ |
| | 4.4 | malic acid | | 1.59 | $1.73 \times 10^{-37}$ |
| | 4.8 | ascorbic acid | | 1.04 | $2.64 \times 10^{-29}$ |
| | 5.4 | lactic acid | | 1.01 | $6.13 \times 10^{-5}$ |
| | 5.7 | acetic acid | | 1.45 | $1.68 \times 10^{-13}$ |
| | 7.7 | maleic acid | | 1.01 | $3.94 \times 10^{-25}$ |
| | 7.8 | citric acid | | 1.30 | $5.90 \times 10^{-39}$ |
| | 8.8 | succinic acid | | 1.35 | $1.22 \times 10^{-19}$ |
| Carotenoids | 9.1 | violaxanthin | 421, 448 | 0.77 | $2.05 \times 10^{-7}$ |
| | 11.2 | neoxanthin | 436, 464 | 0.70 | $2.35 \times 10^{-7}$ |
| | 13.4 | lutein | 400, 422 | 0.92 | $8.33 \times 10^{-8}$ |
| | 14.1 | zeaxanthin | 422 | 0.84 | $7.98 \times 10^{-5}$ |
| | 25.3 | β-cryptoxanthin | 451, 477 | 1.09 | $4.83 \times 10^{-8}$ |
| | 35.6 | *α*-carotene | 446, 474 | 0.87 | $4.38 \times 10^{-5}$ |
| | 38.1 | β-carotene | 436, 464 | 1.00 | $3.98 \times 10^{-14}$ |
| | 39.3 | 9-*cis*-β-carotene | 439, 469 | 0.84 | $1.79 \times 10^{-4}$ |

RT, retention time; VIP, variable importance in the projection.
*p*-Values were analyzed by Duncan's test.
